# Supplementary material for: Comparative Analysis of Mesophilic YqfB-Type Amidohydrolases
Source: Biomolecules. 2022 Oct 16;12(10):1492. doi: 10.3390/biom12101492 (PMC9599836; doi:10.3390/biom12101492)
Supplement: Supplementary file 1 [file biomolecules-12-01492-s001.zip › biomolecules-1929156-supplementary.pdf]

Supplementary information

Comparative Analysis of Mesophilic YqfB-Type Amidohydrolases

Roberta Statkevičiūtė [1,*], Mikas Sadauskas [1], Juta Rainytė [2], Karolina Kavaliauskaitė [1] and Rolandas Meškys [1,*]

[1] Department of Molecular Microbiology and Biotechnology, Institute of Biochemistry, Life Sciences Center, Vilnius University, Sauletekio av. 7, 10257 Vilnius, Lithuania
[2] Department of Eukaryote Gene Engineering, Institute of Biotechnology, Life Sciences Center, Vilnius University, Sauletekio av. 7, 10257, Vilnius, Lithuania
* Correspondence: roberta.statkeviciute@gmc.vu.lt (R.S.); rolandas.meskys@bchi.vu.lt (R.M.)

Synthesis of *N*[4]-modified nucleobases and nucleosides.

First, appropriate amounts of carboxylic acid (Table 2) were activated at room temperature with stirring for 24 hours in 10 ml of ethyl acetate with N,N′-dicyclohexylcarbodiimide (DCC) and N-NHS. Next, appropriate amounts of cytidine, 2′-deoxycytidine and 5-fluorocytidine were left to mix (25-30 °C, 72 h) with activated acid in N,N-dimethylformamide (DMF). The substances were purified on a chromatographic column with chloroform, followed by mixtures of methanol and chloroform, increasing the methanol volume from 2% to 20%. The purification fractions are monitored by TLC analysis (eluent CHCl3:CH3OH; 5:1). The highest concentrations and the purest fractions were combined and the solvents evaporated in a rotary evaporator. Structure, purity and molar mass were confirmed by NMR and HPLC-MS analyses.

N[4]-benzoyl-5-fluorocytidine
Yield 87 mg (17%). HPLC-MS (ESI+): m/z 365.80 $[M+H]^+$; 363.85$[M-H]^-$. UV – λmax1 = 250 nm, λmax3 = 319 nm. 1H (DMSO-d6, 400 MHz) δ = 3.62 (1H, d, J = 12,1 Hz, –CH); 3.74–3.85 (1H, m, –CH); 3.85–3.97 (1H, m, –CH); 4.03 (2H, m, –CH2); 5.09 (1H, d, J = 5.5 Hz, –OH); 5.35 (1H, d, J = 5.9 Hz, –OH); 5.56 (1H, s, –OH); 5.71–5.83 (1H, m, –CH); 7.46–7.58 (2H, m, =CH); 7.60–7.72 (1H, m, –CH); 7.75–8.35 (2H, m, –CH); 8.64 (1H, s, =CH); 10.55 (1H, s, –NH).

N[4]-pivaloyl-5-fluorocytidine
Yield 90 mg (18%). HPLC-MS (ESI+): m/z 345.85 $[M+H]^+$; 343.90 $[M-H]^-$. UV – λmax1 = 219 nm, λmax2 = 318 nm. 1H (DMSO-d6, 400 MHz) δ = 1.06–1.35 (9H, m, –CH3); 3.57–3.68 (1H, m, –CH); 3.74–3.79 (1H, m, –CH); 3.88–3.94 (1H, m, –CH); 3.96–4.04 (2H, m, –CH2); 5.08 (1H, d, J = 5.3 Hz, –OH); 5.36 (1H, d, J = 5.2 Hz, –OH); 5.57 (1H, s, –OH); 5.69–5.74 (1H, m, –CH); 8.71 (1H, s, =CH). 13C BMR (DMSO-d6, 101 MHz) δ = 27.17; 27.48; 38.29; 60.58; 69.44; 74.68; 84.44; 89.72; 126.30; 154.01; 170.72.

Synthesis of acetylated 5-fluoroisocytosines
1.92 g (40 mmol) of NaH (60% dispersion in mineral oil) was dissolved in diethyl ether (280 ml), followed by the addition of ethyl formate (3.22 ml; 40 mmol) and ethyl fluoroacetate (3.86 ml; 40 mmol) and stirred for 19 hours at room temperature. The reaction mixture was concentrated in a rotary evaporator to give a yellow precipitate.The guanidine hydrochloride salt, 11.44 g (120 mmol) was neutralised with NaOEt.

2.5 M solution, which was previously prepared by dissolving 2.76 g (120 mmol) sodium in ethanol. The mixture was stirred on a magnetic stirrer for 30 minutes and then filtered by vacuum filtration. The sodium ethyl-(2*E*)-2-fluoro-3-hydroxy-2-propenoate, previously obtained, was dissolved in the filtrate and stirred and heated at 90 °C for 20 hours. The resulting material was filtered, acidified with concentrated HCl acid (pH 5.0) and purified on a chromatographic column by the reversed-phase method (eluent to water) with methanol, increasing the volume ratio of methanol from 0 to 20%. The purification fractions are monitored by thin-layer chromatographic analysis (eluent - CHCl3:CH3OH; 5:1). The highest concentrations and the purest fractions are combined and the solvents are evaporated in a rotary evaporator. 1.80 g of 5-fluoroisocytosine powder was obtained (35% yield). The structure and molecular weight of the compound are confirmed by HPLC-MS and NMR. HPLC-MS (ESI+): m/z 130.05 $[M+H]^+$; 128,05 $[M-H]^-$. UV – λ max = 263 nm. 1H (DMSO-d6, 400 MHz) δ = 6.69 (2H, s, -NH2); 7.64 (1H, s, =CH). 13C NMR (DMSO-d6, 101 MHz) δ = 137.03; 142.36; 153.43 153.79. *N*-acylation was carried out as described previously [1].

Table S1. DNA and amino acid sequences of YqfB-type proteins used in this study.

Organism	Gene and protein sequences
Buttiauxella agrestis	Gene:<br>ATGAACGACATAACATTTTTCCAACGCTTTGAGAGCGATATCGTTGCCGGG<br>CGCAAAACCATTACGCTGCGCGATGTTTCGGAGTCGCACTTCATCCCAGGC<br>CAACAGTTGCGCGTCGGGCGTTATGAAGATGACGTTTACTTTTGTACCATTG<br>AAGTCGTGAGCGTGACCCCCGTGGCGCTTGAAAAGCTGACCGACGAGCAC<br>GCGCGTCAGGAAAATATGACACTGCCGGAACTCAAAGAAGTGATTGGTGA<br>AATTTACCCCGGTTTGACGGAGCTTTACGCGATTGTTTTTAGATTGCTGACA<br>AAGGGCGATAAACGGGTTTTGTAG<br><br>Protein (WP_172894188):<br>MNDITFFQRFESDIVAGRKTITLRDVSESHFIPGQQLRVGRYEDDVYFCTIEVVSVT<br>PVALEKLTDEHARQENMTLPELKEVIGEIYPGLTELYAIVFRLLTKGDKRVL*
Cronobacter universalis	Gene:<br>ATGGGCCGTAATTTTGGGAGAGACGCAATGAAAAACGACATCACATTCTAC<br>GCCCGTTTCCAGCAGGATATTCTCGCGGGAACCAAAACGATCACCATCCGT<br>GACGGCAGCGAGGCGCACTTCACCCCAGGCCAGCGGCTGCGCACCGGCCG<br>TTATGAAGATAATGGCTACTTCTGCACGCTTGAAGTGCTTAGCGTGACGCCG<br>GTCACTCTGGCAGAGCTGAACGACGAACACGCGCGTCAGGAGAACATGAC<br>GCTGGCTGAACTTAAACACGTCATTACAGAGATTTACCCGGGGCTTAATGA<br>GTTCTATGTGATTGCTTTTAAAAAGATTGATGAATAA<br><br>Protein (CCK15589):<br>MGRNFGRDAMKNDITFYARFQQDILAGTKTITIRDGSEAHFTPGQRLRTGRYED<br>NGYFCTLEVLSVTPVTLAELNDEHARQENMTLAELKHVITEIYPGLNEFYVIAFK<br>KIDE*
Klebsiella pneumoniae<br>KV-3	Gene:<br>ATGCAGGCCAATGACATTACCTTTTTTCAGCGTTTTCAGGACGACATCCTCG<br>CCGGGCGCAAAACCATCACCATTCGCGATGCAGCGGAGTCGCACTTTAAGC<br>CCGGCGATGTGCTGCGCGTTGGGCGCTATGAAGATGACGGCTATTTTTGCAC<br>CATTGCCGTGACCGCGACCTCGACGGTGACGCTCGATACGCTGACCGAACA<br>GCATGCGCAACAGGAGAATATGACTCTCGGGCAGCTGCGGCAGGTCATCA<br>GCGACATCTATCCTGGCGAGAGCCAGTTTTATGTCATTGAATTCAAAACGCT<br>TTAA<br><br>Protein (WP_251891168):<br>MQANDITFFQRFQDDILAGRKTITIRDAAESHFKPGDVLRVGRYEDDGYFCTIAV<br>TATSTVTLDTLTEQHAQQENMTLGQLRQVISDIYPGESQFYVIEFKTL*
Shewanella loihica	Gene:<br>ATGACCCCGTTAACCTATGATGGCAAGATCACCTTCTTCGAGCGATTCGAA<br>GCCGATATCCTCAGCGGAGCGAAAACCATCACCCTCAGGGATGAGAGCGA<br>GTCGCACTTTCGCGTCGGTGACCGCTTGTCGGTATCCACCTTTGAAACCGGG<br>CGACACTTTTGCGATATTCAGGTGGTGCGGGTCGAAGAGCTTGCCTTCGAG<br>GCATTAAACTCCCGGCACGCCGTGCAGGAGAATATGACGCTTGAGCAGTTA<br>AAGGCGGTGATCACCGAGATATATGGCGAGATTGATACCCTCTATCTTATC<br>GAGTATCGTCTGCTCTAG<br><br>Protein (WP_011865293):<br>MTPLTYDGKITFFERFEADILSGAKTITLRDESESHFRVGDRLSVSTFETGRHFCDI<br>QVVRVEELAFEALNSRHAVQENMTLEQLKAVITEIYGEIDTLYLIEYRLL*

Table S2. List of primers used in this study.

Organism	Primer name	Sequence (5′ → 3′)
Buttiauxella agrestis	BagYqfB_F	AGAAGGAGATATAACTATGAACGACATAACATTTTT
	BagYqfB_R	GTGGTGGTGATGGTGATGGCCCAAAACCCGTTTATCG
Cronobacter universalis	CunYqfB_F	AGAAGGAGATATAACTATGGGCCGTAATTTTGG
	CunYqfB_R	GTGGTGGTGATGGTGATGGCCTTCATCAATCTTTTTAAAAGC
Klebsiella pneumonia	KpnYqfB_F	AGAAGGAGATATAACTATGCAGGCCAATGA
	KpnYqfB_R	GTGGTGGTGATGGTGATGGCCAAGCGTTTTGAATTCAA
Shewanella loihica	SloYqfB_F	AGAAGGAGATATAACTATGACCCCGTTAACC
	SloYqfB_R	GTGGTGGTGATGGTGATGGCCGAGCAGACGATACTC

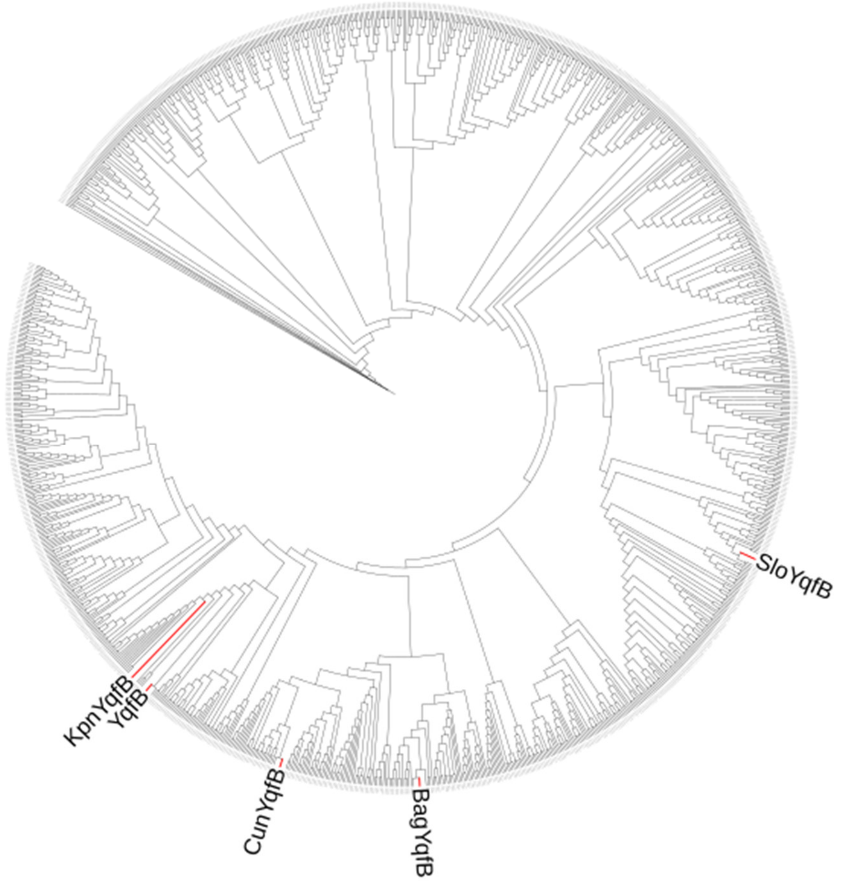

Figure S1. Phylogenetic tree of ASCH domain-containing proteins obtained from protein BLAST search results (over 1000 sequences). The multiple sequence alignment was performed by applying Clustal Omega algorithm and generated phylogenetic tree was visualised with the iTOL tool. Highlighted are the proteins used in this study.

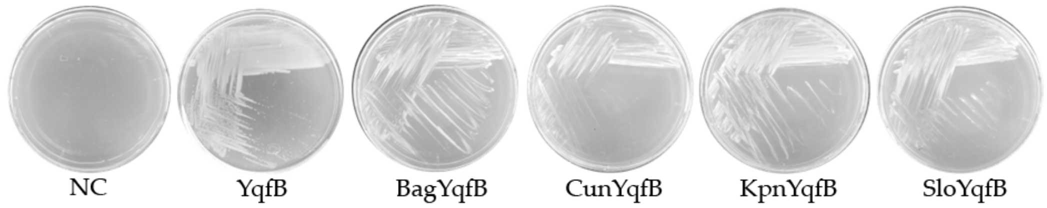

Figure S2. Growth of *E. coli* HMS174 Δ*pyrF* Δ*yqfB* cells on minimal media supplemented with ac4C. NC – negative control, cells transformed with the pET21b vector without an insert; YqfB – cells producing previously characterized amidohydrolase YqfB from *E. coli* were used as a positive control.

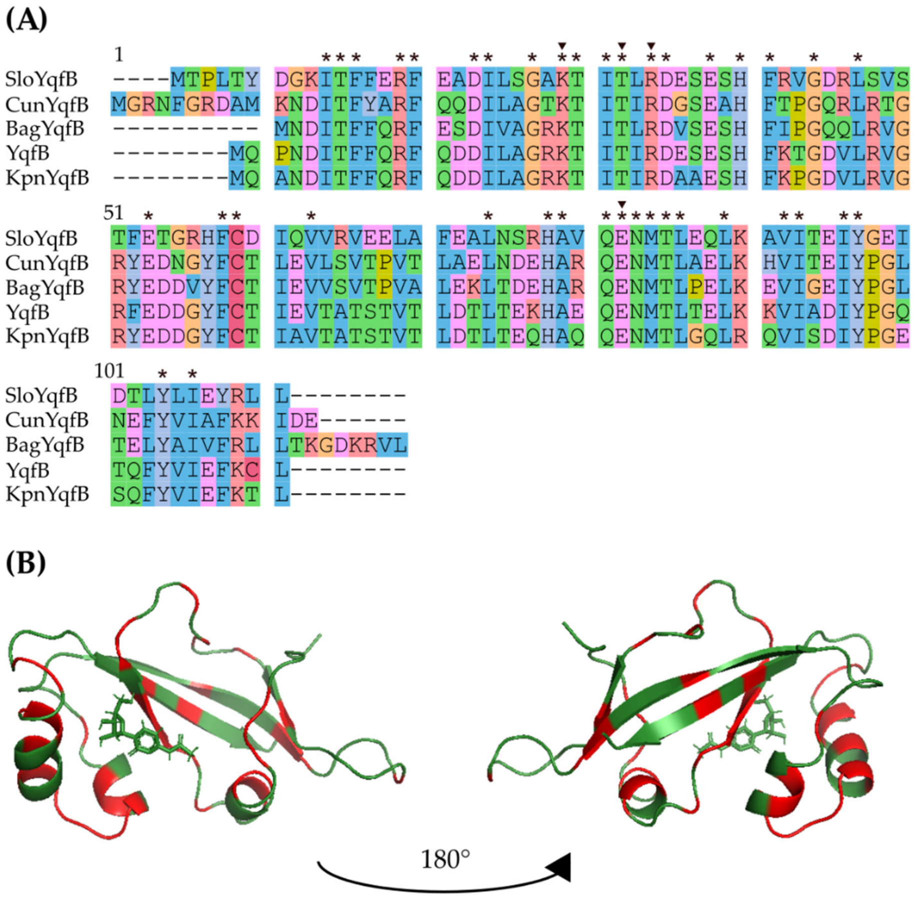

Figure S3. Analysis of conserved regions of YqfB-type amidohydrolases. (A) Multiple protein sequence alignment was performed using the Clustal Omega tool and visualized with SeaView4. The conserved and catalytic amino acids of YqfB are marked with asterisks and triangles, respectively; (B) predicted 3D model of E. coli YqfB where conserved residues of YqfB analogues used in this study are highlighted in red and variable regions in green. The visualization was performed using PyMOL Molecular Graphics System Version 2.5.4.

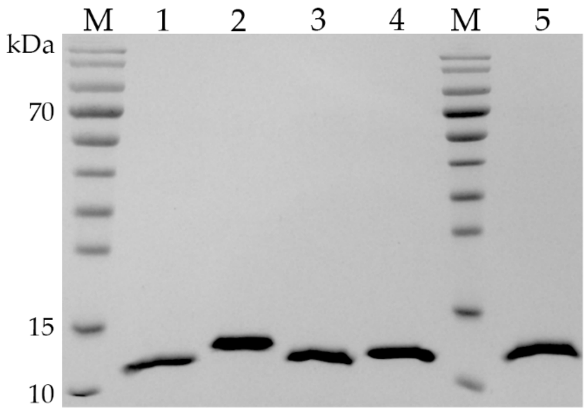

Figure S4. Profiles of purified YqfB analogues in SDS-PAGE, where M – PageRuler Prestained Protein Ladder; 1 - BagYqfB; 2 – CunYqfB; 3 – KpnYqfB; 4 – SloYqfB; 5 – YqfB.

N^4-acetylcytosine

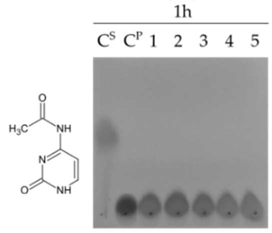

N^4-acetylcytidine

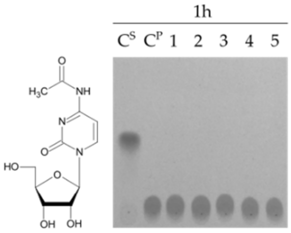

N^4-acetyl-2'-deoxycytidine

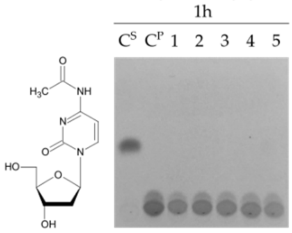

N^2-acetylisocytosine

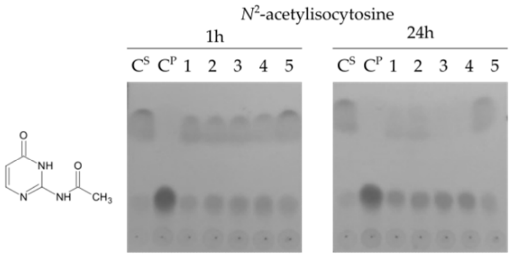

N^2-pivaloylisocytosine

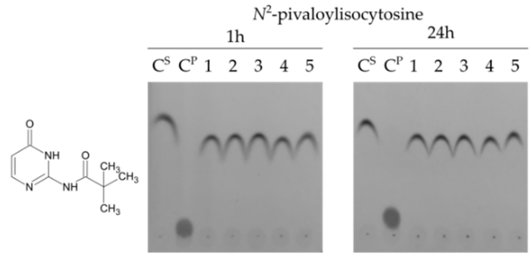

N^4-benzoylcytidine

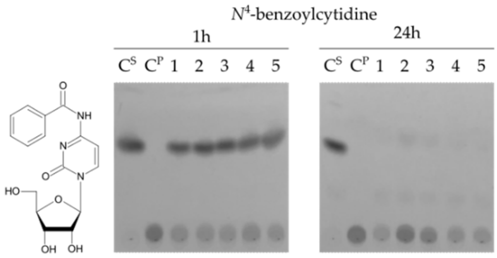

N^4-isobutyryl-2'-deoxycytidine

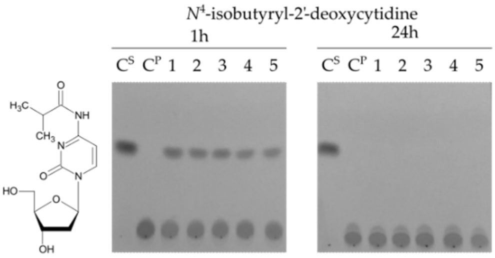

N^4-hexanoyl-2'-deoxycytidine

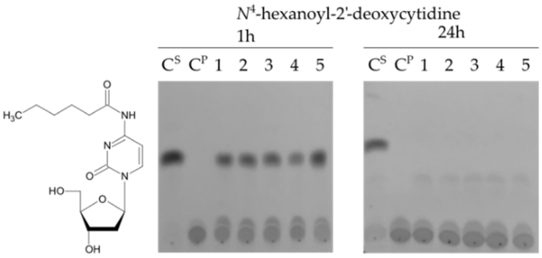

N^4-nicotinoyl-2'-deoxycytidine

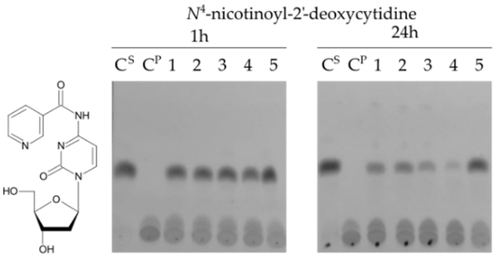

N^4-(3-acetyl-benzoyl)-2'-deoxycytidine

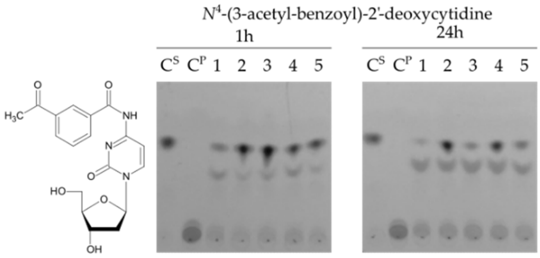

N^4-(4-acetyl-benzoyl)-2'-deoxycytidine

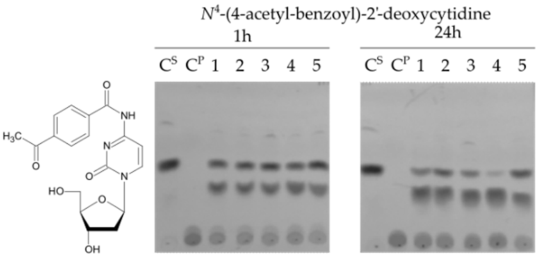

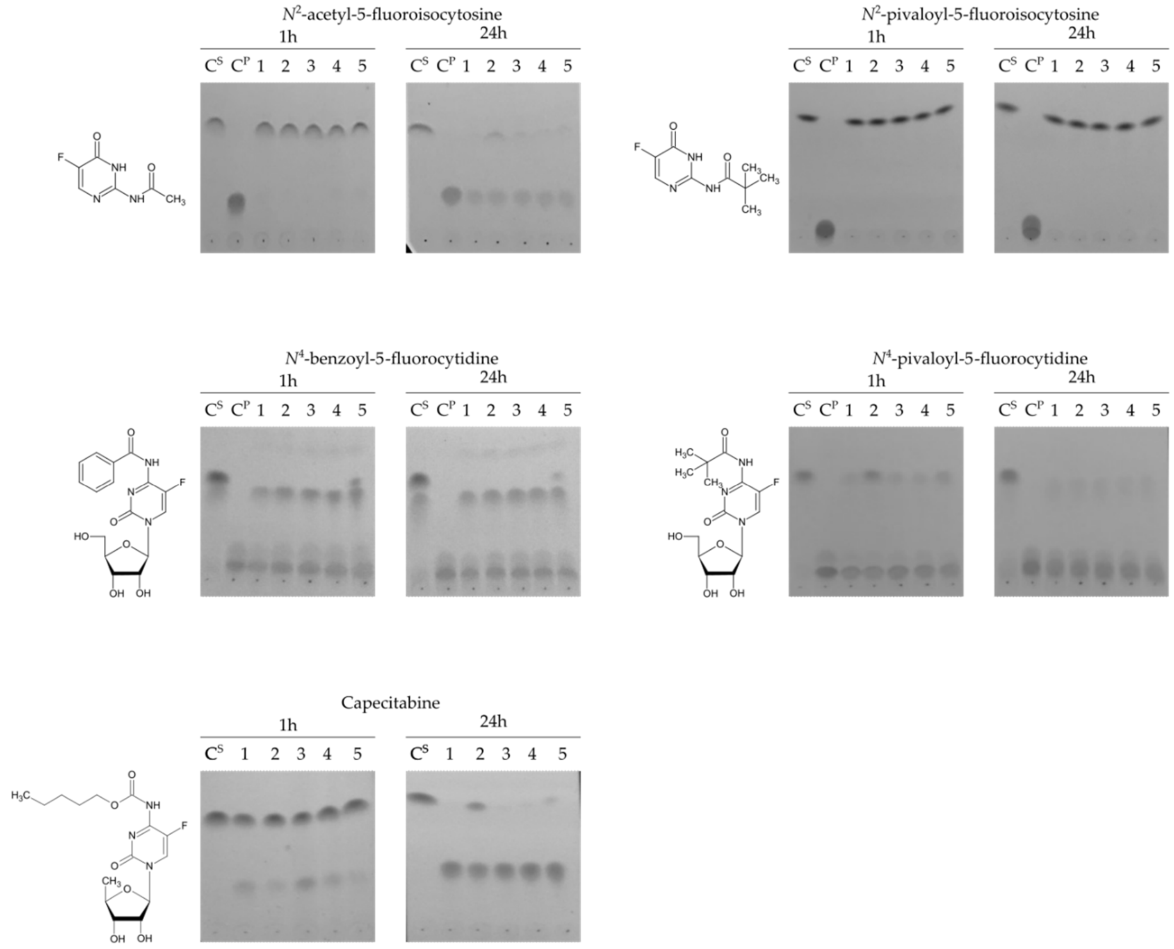

Figure S5. Qualitative enzymatic activity analysis of amidohydrolases. C^{S} – substrate control; C^{P} – product control; 1 – YqfB; 2 – BagYqfB; 3 – CunYqfB; 4 – KpnYqfB; 5 – SloYqfB.

```
    SignalP-NN euk predictions are as follows:

  # name                Cmax  pos ?  Ymax  pos ?  Smax  pos ?  Smean ?  D     ?
  Sequence                  0.108  58  0.110  12  0.132   1  0.100   0.105 N  0.450      SignalP-noTM

    SignalP output is explained at https://services.healthtech.dtu.dk/services/SignalP-4.1/output.php

##########################################################################################

Name:  Sequence         Length:  110
GMQPNDITFFQRFQDDILAGRKTITIRDESESHFKTGDVLRVGRFEDDGYFCTIEVTATSTVTLDTLTEKHAEQENMTLT      80
ELKKVIADIYPGQTQFYVIEFKCLHHHHHH
...........................................................................N....      80
..............................                                                      160

(Threshold=0.5)
----------------------------------------------------------------------
SeqName      Position  Potential   Jury    N-Glyc
                                 agreement result
----------------------------------------------------------------------
Sequence       76 NMTL   0.6858     (9/9)   ++
----------------------------------------------------------------------
```

Figure S6. Predicted YqfB *N*-glycosylation sites in yeast using NetNGlyc – 1.0 server [2].

References

1. Stanislauskienė, R.; Laurynėnas, A.; Rutkienė, R.; Aučynaitė, A.; Tauraitė, D.; Meškienė, R.; Urbelienė, N.; Kaupinis, A.; Valius, M.; Kaliniene, L.; et al. YqfB Protein from *Escherichia coli*: An Atypical Amidohydrolase Active towards N4-Acylcytosine Derivatives. *Sci. Rep.* **2020**, *10*, 788. https://doi.org/10.1038/s41598-020-57664-w.
2. Gupta, R.; Brunak, S. Prediction of Glycosylation across the Human Proteome and the Correlation to Protein Function. In *Pacific Symposium on Biocomputing 2002, Proceedings of the Pacific Symposium, Kauai, Hawaii, USA, 3–7 January 2002*; Altman, R.B.; Dunker, A.K.; et al., Eds.; World Scientific: Singapore, 2002; 310–322.
